# Individual Amino Acid Supplementation Does Not Enhance Short-Term Proliferation of Selected Cancer Cell Lines In Vitro: Potential Implications for Nutritional Support in Cancer Cachexia

**DOI:** 10.3390/nu18132125

**Published:** 2026-07-01

**Authors:** Walburga Dieterich, Rashmita Pradhan, Abdulhadi Suwandi, Rabia Ülkü Korkmaz, Markus F. Neurath, Yurdagül Zopf

**Affiliations:** 1 Department of Medicine 1, Friedrich-Alexander-University Erlangen-Nürnberg, 91054 Erlangen, Germany; rashmita.pradhan@uk-erlangen.de (R.P.); abdulhadi.suwandi@uk-erlangen.de (A.S.); rabia.korkmaz@uk-erlangen.de (R.Ü.K.); markus.neurath@uk-erlangen.de (M.F.N.); yurdaguel.zopf@uk-erlangen.de (Y.Z.); 2 Hector-Center for Nutrition, Exercise and Sports, Department of Medicine 1, Friedrich-Alexander-University Erlangen-Nürnberg, 91054 Erlangen, Germany; 3 Deutsches Zentrum Immuntherapie (DZI), University Hospital Erlangen, Friedrich-Alexander-University Erlangen-Nürnberg, 91054 Erlangen, Germany

**Keywords:** cancer cachexia, amino acid supplementation, tumor cell proliferation, cell metabolism, apoptosis, sarcopenia, nutritional support

## Abstract

**Background**: Cancer-related cachexia is primarily characterized by systemic inflammation and progressive muscle wasting, which is why a high-protein diet (from 1.2 to 1.5 g/kg/day) is commonly recommended. However, concerns remain that an excessive supply of amino acids could promote tumor growth due to the metabolic flexibility of cancer cells, thereby favoring proliferation and survival. Systematic evidence addressing these concerns under controlled conditions for various types of cancer cells remains limited and inconclusive. **Methods**: We investigated the short-term effects of all 20 amino acids at both moderate (2×) and high (10×) concentrations to evaluate three key oncological endpoints in four human cancer cell lines: MDA-MB-231 (breast), HT29 (colorectal), PC3 (prostate), and PANC-1 (pancreatic). Cell proliferation was assessed by BrdU incorporation, metabolic activity by WST-1 assay, and apoptosis signaling by caspase-3/7 activity measurement. **Results**: Amino acid supplementation was not associated with a significant change in proliferation at either concentration across all four cell lines studied. Metabolic activity showed only minor variations throughout, with PC3 cells exhibiting slightly greater variability, although this did not reach statistical significance. Caspase-3/7 activity remained largely unchanged under all conditions; however, high-concentration lysine induced an approximately 2.5-fold increase in PANC1 cells, which was not statistically significant. **Conclusions**: These findings suggest that short-term exposure to individual amino acids, even at supraphysiological conditions, does not acutely enhance proliferative activity in the cancer cell lines studied, supporting the rationale for adequate protein and amino acid intake in patients with cancer cachexia.

## 1. Introduction

Proper energy and nutrient intake is critical for cancer patients, particularly those affected by cancer-related cachexia. Cancer cachexia affects about one-third of all cancer patients, with the highest prevalence observed in lung, pancreatic, esophageal, stomach, and liver cancers. Cancer cachexia significantly impairs physical functioning and quality of life and is associated with increased mortality [1,2]. This condition is characterized by progressive weight loss, muscle wasting, and systemic inflammation, resulting in a highly catabolic state. To counteract protein breakdown, maintain muscle mass, and improve overall outcomes in cancer patients, nutritional therapy generally recommends a high-protein diet, with an intake of approximately 1.2–1.5 g of protein per kilogram of body weight each day [3,4,5].

Adequate protein intake is clinically important in cancer patients. Clinical studies have already demonstrated that certain mixtures of essential amino acids improve muscle mass and strength in patients with cachexia, and in vitro data also suggest that these amino acid mixtures inhibit tumor proliferation [6]. The supplementation with branched-chain amino acids improved the survival rate of mice with Lewis lung cancer and alleviated cancer induced cachexia [7]. Furthermore, in the colon-26 carcinoma mouse model, the preventive effect of branched-chain amino acids against muscle atrophy was further enhanced by the addition of di-alanine [8]. However, concerns remain that increased amino acid availability could promote tumor growth. Tumor cells exhibit considerable metabolic flexibility, utilizing amino acids not only for protein and nucleotide synthesis but also as key substrates for energy production, biosynthesis, and signaling pathways that support growth and survival [9]. Evidence regarding the impact of amino acid supplementation on cancer metabolism and proliferation remains inconclusive. Some studies suggest that specific amino acids may enhance tumor growth [6,10,11], whereas others indicate that amino acid deprivation or imbalance can induce metabolic stress and trigger programmed cell death under certain conditions [6,10,12,13,14]. Glutamine is the most abundant amino acid and plays a central role in cellular growth and metabolism; many cancer cells are highly dependent on the availability of glutamine. When there is an excess of cellular amino acids, nutrient-sensing pathways activate mTORC1, thereby promoting protein synthesis and cell growth. An amino acid deficiency, particularly a glutamine deficiency, also activates the stress sensor GCN2, which in turn suppresses mTORC1 activity, leading to the inhibition of anabolic processes and cell growth [15]. Knowledge of amino acid metabolism in cancer cells is therefore essential for developing therapeutic strategies.

However, comparative analyses of amino acid-mediated metabolic and apoptotic responses across different cancer cell lines remain limited. In particular, no systematic study has evaluated the effects of all 20 amino acids across multiple tumor cell lines under identical experimental conditions. Studying individual amino acids allows the identification of specific effects that could be masked in complex protein mixtures, providing a clearer understanding of how amino acid availability influences proliferation, metabolism, and apoptotic signaling.

Here, we investigated the impact of all 20 amino acids, at moderate and supraphysiological concentrations, on short-term proliferation, metabolic activity, and caspase-3/7 mediated apoptosis in four cancer cell lines. In addition to investigating concentrations that may reflect increased availability of amino acid during nutritional support, we deliberately used supraphysiological concentrations to explore whether an extreme substrate load induces metabolic stress and alters the behavior of cancer cells. MDA-MB-231 (breast), HT29 (colorectal), PC3 (prostate), and PANC-1 (pancreatic) cells were used because they represent clinically highly relevant tumors of various tissue origins with distinct genetic backgrounds. MDA-MB-231, PC3, and PANC-1 exhibit high invasiveness, migratory capacity, and a high potential for metastasis, whereas HT29 is less invasive. This reflects some of the biological heterogeneity characteristics of human cancers. We hypothesized that increased amino acid availability, including supraphysiological concentrations, would not significantly stimulate tumor cell proliferation. The present study therefore aimed to explore whether amino acid supplementation exerts measurable effects on proliferation, metabolic activity, and apoptosis-related signaling in different cancer cell lines.

## 2. Materials and Methods

### 2.1. Cell Culture

MDA-MB-231 (breast cancer adenocarcinoma; ATCC, Manassas, VA, USA; RRID: CVCL_0062), PC3 (prostate cancer; ATCC, Manassas, VA, USA), HT29 (colorectal cancer; ATCC HTB-38, Manassas, VA, USA; Lot: 17A026), and PANC-1 (pancreatic cancer; Sigma-Aldrich, Munich, Germany) cells were maintained in Dulbecco’s Modified Eagle Medium (DMEM GlutaMAX™; Gibco, cat. no. 10566, Thermo Fisher Scientific, Waltham, MA, USA) supplemented with 10% fetal calf serum (FCS Superior; Merck KGaA, Darmstadt, Germany) and 100 IU/mL penicillin/100 µg/mL streptomycin (Gibco, Thermo Fisher Scientific, Waltham, MA, USA). Cells were cultured at 37 °C in a humidified atmosphere containing 5% CO_2_ and used for up to 30 passages. All cell cultures tested negative for mycoplasma contamination.

### 2.2. Amino Acid Treatment

For all experiments, cells were seeded in 96-well plates at 5–8 × 10^3^ cells per well and cultured overnight at 37 °C in a humidified 5% CO_2_ atmosphere. To reduce baseline amino acid levels, cells were starved for 24 h in starvation medium consisting of a 1:10 mixture of DMEM GlutaMAX™ and DMEM without amino acids (Genaxxon Bioscience, cat. no. C4150, Ulm, Germany), supplemented with 0.1% FCS and 100 IU/mL penicillin/100 µg/mL streptomycin. This medium contained approximately 10% of the amino acids normally present in standard DMEM.

Following starvation, cells were switched to recovery medium consisting of a 1:5 mixture of DMEM GlutaMAX™ and DMEM without amino acids (Genaxxon, C4150), supplemented with 2% FCS and 100 IU/mL penicillin/100 µg/mL streptomycin. This recovery medium contained approximately 20% of the amino acids normally present in standard DMEM, providing a low baseline that allowed for controlled supplementation of individual amino acids (Table 1). Cells were cultured under these conditions for 24 h.

Individual amino acids were added at twofold (2×) or tenfold (10×) their basal DMEM concentrations, representing moderate and high levels commonly used in cell culture. All amino acids were purchased as powder (Carl ROTH, Karlsruhe, Germany) and dissolved in a 0.9% sodium chloride solution (pH 7.4), except for tyrosine, which was dissolved at a pH of 10.2. Concentrations were selected based on the standard formulation of DMEM/GlutaMAX™ medium and RPMI1640 (Gibco, cat. no 11875, Thermo Fisher Scientific, Waltham, MA, USA).

### 2.3. Cell Proliferation Assay

Cell proliferation was assessed using a 5-bromo-2′-deoxyuridine (BrdU) incorporation assay (Cell Proliferation ELISA, Roche Applied Science, Penzberg, Germany). After 24 h of starvation, cells were cultured in recovery medium containing 2% FCS and 20% of the standard amino acid concentration, and supplemented with individual amino acids at twofold (2×) and tenfold (10×) concentrations. After 6 h of amino acid treatment, 10 µM BrdU was added, and cells were incubated for an additional 18 h, resulting in a total treatment of 24 h.

Cells were then lysed, and BrdU incorporation was quantified according to the manufacturer’s instructions. Each condition was tested in duplicates, and experiments were repeated independently three times. Data are expressed relative to the control group.

### 2.4. Metabolic Activity Assay

Cellular metabolic activity was assessed by measuring mitochondrial dehydrogenase activity using the water-soluble tetrazolium salt-1 (WST-1) assay according to the manufacturer’s instructions (cat. no. 11644807001, Roche Diagnostics GmbH, Mannheim, Germany).

Briefly, after 24 h of amino acid treatment, WST-1 reagent was added and incubated for 1 h at 37 °C. Absorbance was measured at 450 nm with a reference wavelength of 600 nm using a microplate reader (NOVOStar, BMG Labtech, Offenburg, Germany). Data are expressed relative to the control group.

### 2.5. Caspase-3/7 Apoptosis Assay

Caspase-3/7 activity was measured using the EnzCheck™ Caspase-3 Activity Assay Kit (E13184, Thermo Fisher Scientific, Invitrogen, Waltham, MA, USA) according to the manufacturer’s instructions. Cells were seeded at equal densities for all conditions.

Briefly, cells were cultured and treated with individual amino acids for 24 h. Subsequently, cells were washed with phosphate-buffered saline (PBS) and lysed using the supplied lysis buffer. Caspase-3/7 activity was determined by incubating cell lysates with a rhodamine-labelled substrate, which is cleaved by active caspase-3/7. Fluorescence was measured after 30 min at 489/520 nm (excitation/emission). Caspase activity was expressed as relative fluorescence units (RFU) per well.

### 2.6. Statistical Analysis

All experiments were performed in duplicate and repeated independently three times (n = 3). Data are presented as mean ± SD. Statistical significance was evaluated using an unpaired *t*-test with Welch correction. Multiple comparisons were corrected using a false discovery rate (FDR) approach with the two-stage step-up method of Benjamini, Krieger, and Yekutieli.

## 3. Results

### 3.1. Amino Acid Supplementation Does Not Enhance Cancer Cell Proliferation (Figure 1)

**Figure 1:**
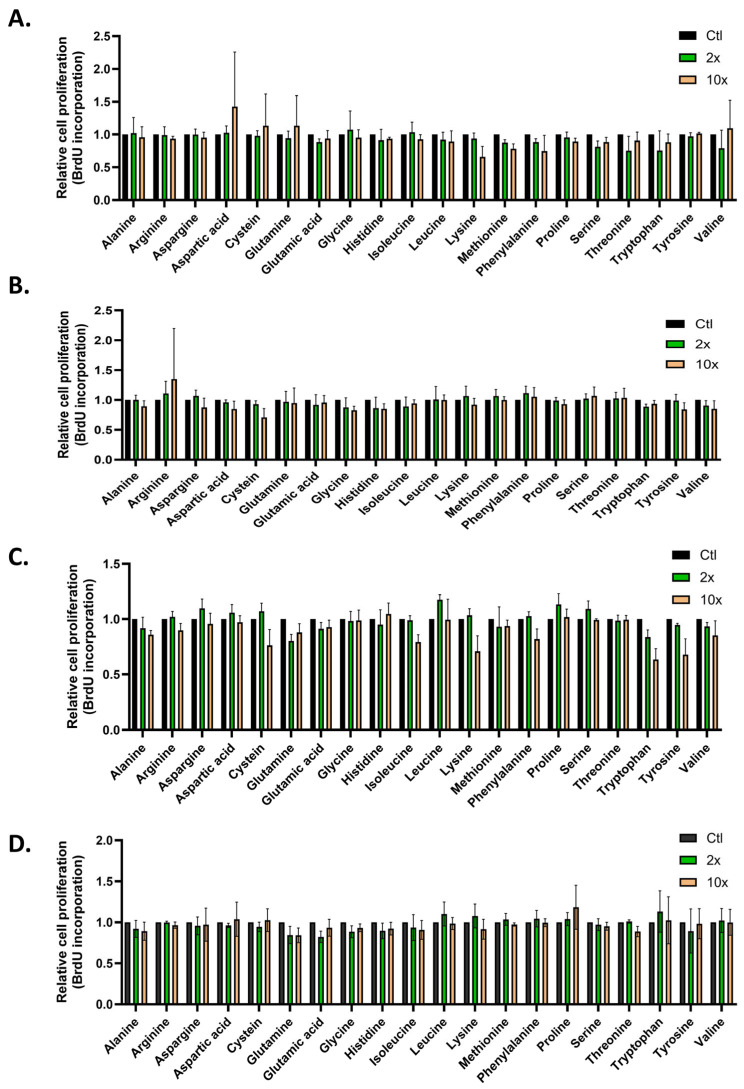
Effect of amino acids supplementation on cancer cell proliferation after 24 h. (**A**) HT29, (**B**) MDA-MB-231, (**C**) PC3, and (**D**) PANC-1 cells were serum-starved in medium containing 0.1% FCS for 18–24 h and then treated with individual amino acids at the indicated concentrations (Ctl: control; 2× and 10×: two- and tenfold amino acid concentrations, respectively). Cell proliferation was measured by BrdU incorporation and expressed relative to the medium control. Data represent the mean ± SD from three independent experiments (n = 3 per cell line). Statistical analysis was performed using unpaired *t*-test with Welch correction and multiple comparisons with false discovery rate (FDR).

To address concerns that amino acid supplementation could stimulate tumor growth, we first examined its effects on cancer cell proliferation. Overall, supplementation at both moderate (2×) and high (10×) concentrations had no significant effect on cell proliferation across all four cancer cell lines tested. Proliferation was assessed using the BrdU incorporation assay (Figure 1).

In HT29 (Figure 1A), amino acid supplementation had no significant effect on proliferation, although 10× aspartic acid showed a slight non-significant increase in BrdU incorporation. MDA-MB-231 cells (Figure 1B) demonstrated no consistent changes in proliferation across conditions. In PC3 cells (Figure 1C), high amino acid concentrations (10×) were associated with modest, non-significant decreases in proliferation, while PANC-1 cells (Figure 1D) remained unaffected under all conditions tested.

Importantly, none of the individual amino acids significantly increased proliferation in any cell lines. These results suggested that amino acid supplementation, even at higher concentrations, does not appear to enhance proliferation activity under the conditions tested.

### 3.2. Amino Acid Supplementation Does Not Alter Metabolic Activity (Figure 2)

**Figure 2:**
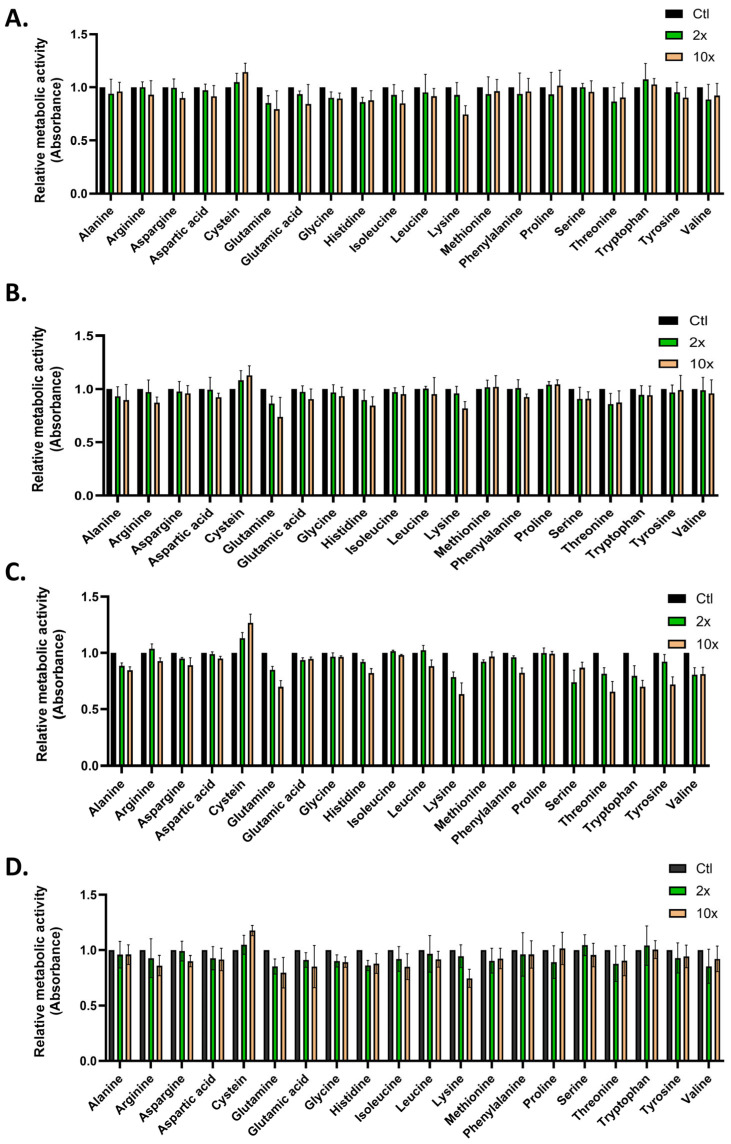
Effect of amino acids supplementation on the metabolic activity of cancer cells after 24 h. (**A**) HT29, (**B**) MDA-MB-231, (**C**) PC3, and (**D**) PANC-1 cells were treated with individual amino acids at the indicated concentration. (Ctl: control; 2× and 10×) for 24 h. Metabolic activity was assessed using WST-1 colorimetric assay and absorbance was measured at 450 nm with a reference wavelength of 600 nm. Data represent the mean ± SD from three independent experiments (n = 3 per cell line). Statistical analysis was performed using unpaired *t*-test with Welch correction and multiple comparisons with false discovery rate (FDR).

We next assessed the effect of amino acids on cellular metabolic activity using the WST-1 assay, which measures mitochondrial dehydrogenase activity and detects early changes in cell vitality (Figure 2). In all four cancer cell lines, amino acid supplementation resulted in only minor variations in metabolic activity after 24 h of treatment.

Cysteine at the highest concentration (10×) was associated with a slight increase in metabolic activity across cell lines. In contrast, lysine (10×) treatment showed a non-significant trend toward reduced metabolic activity. High concentrations of glutamine and lysine supplementation also caused modest reductions in metabolic activity in all tumor cell lines (Figure 2A–D).

Among the cell lines, the PC3 cell (Figure 2C) showed slightly greater variations in metabolic activity under the different amino acid conditions. In addition to lysine and glutamine, threonine, tryptophan, and tyrosine caused minor changes in this cell line. However, none of these differences were statistically significant, and no consistent concentration-dependent effects were detected. Furthermore, metabolic activity in PANC-1 cells (Figure 2D) remained largely unchanged across all amino acids and concentrations tested.

These findings, which are consistent with the proliferation results, suggest that amino acid supplementation under the conditions selected in this study does not enhance cancer cell metabolic activity.

### 3.3. Amino Acid Supplementation Does Not Induce Apoptosis-Related Caspase-3/7 Activation (Figure 3)

**Figure 3:**
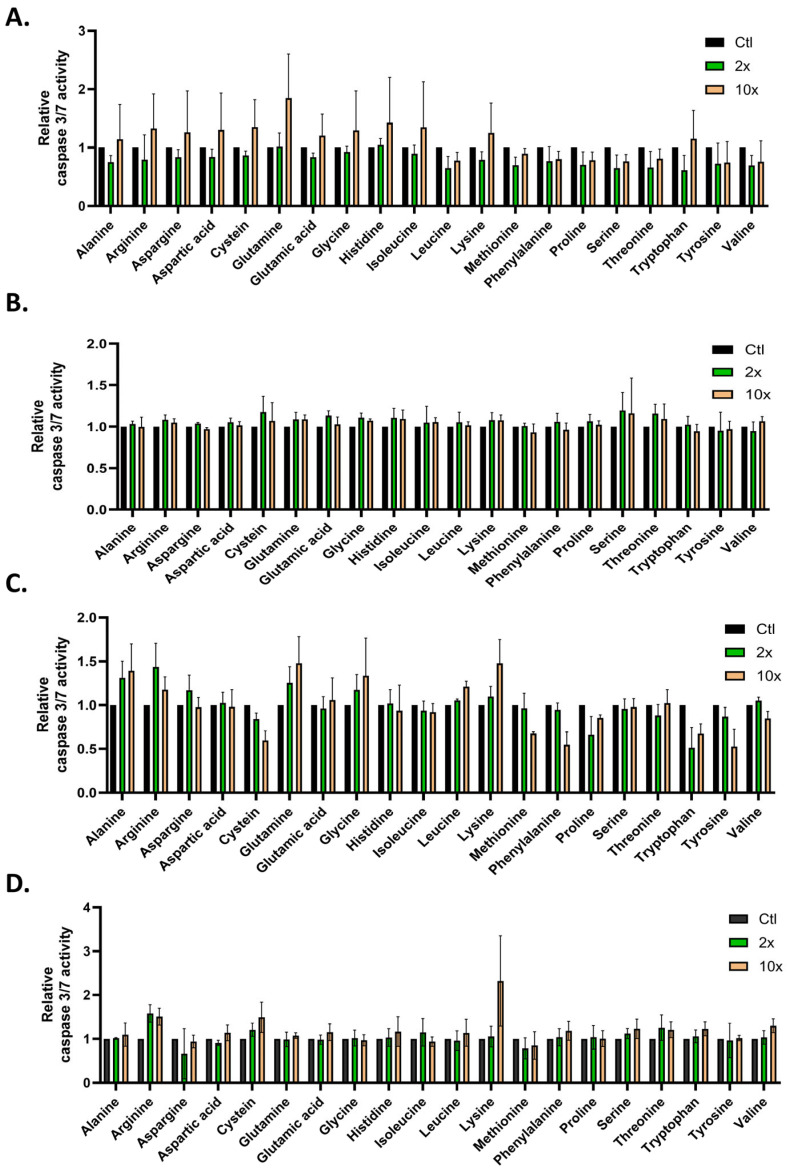
Effect of amino acids supplementation on caspase-3/7 activity in cancer cell lines after 24 h. (**A**) HT29, (**B**) MDA-MB-231, (**C**) PC3, and (**D**) PANC-1 cells were treated with individual amino acids at indicated concentrations (Ctl: control; 2× and 10×) for 24 h. Apoptosis was measured by caspase 3/7 activity using EnzCheck Caspase-3 Activity Assay Kit with excitation/emission wavelengths at 490/570 nm. Data represent the mean ± SD from three independent experiments (n = 3 per cell line). Statistical analysis was performed using unpaired *t*-test with Welch correction and multiple comparisons with false discovery rate (FDR).

We next assessed whether amino acid supplementation induces programmed cell death by measuring caspase-3/7 activity (Figure 3). In HT29 cells (Figure 3A), high glutamine supplementation (10×) led to a slight increase in caspase-3/7 activity, while several amino acids caused a marginal decrease in caspase activity. MDA-MB-231 cells (Figure 3B) showed only minimal variations in caspase-3/7 activity following amino acid supplementation. PC3 cells (Figure 3C) exhibited modest variability, with slight increases in alanine, arginine, glutamine, glycine, and lysine, and slight decreases in cysteine, methionine, phenylalanine, tryptophan, and tyrosine. In PANC-1 cells (Figure 3D), arginine, and especially lysine at high concentrations, induced an approximately 2.5-fold increase in caspase-3/7 activity; however, none of the changes reached statistical significance.

Overall, amino acid supplementation did not significantly induce caspase-3/7 activity in any of the tested cell lines. Together with the proliferation and metabolic data, these findings suggest that amino acid supplementation did not measurably affect proliferation, metabolic activity, or apoptosis-related signaling under the experimental conditions tested. However, the relatively small number of three independent biological replicates does not allow for definitive conclusions regarding the absence of an effect.

## 4. Discussion

In routine oncological practice, adequate protein intake is essential, particularly for patients affected by cancer-associated cachexia or sarcopenia, conditions characterized by muscle wasting, systemic inflammation, and metabolic dysregulation [3]. However, cancer patients frequently consume suboptimal amounts of protein due to treatment-related symptoms, including nausea, vomiting, constipation, and altered taste, which could lead to or exacerbate muscle wasting [16,17]. Clinical guidelines recommend protein intakes of approximately 1.2–1.5 g/kg body weight per day to preserve skeletal muscle mass in cancer patients [3,4]. Despite this well-established clinical rationale, concerns remain that increased protein or amino acid availability could support tumor growth. This apparent contradiction between clinical necessity and concerns regarding oncological safety represents the fundamental question addressed in the current study.

Our findings indicate that short-term supplementation with individual amino acids at concentrations up to tenfold higher than those used in standard culture medium did not significantly affect acute proliferation, metabolic activity, or caspase-3/7 activation across four different cancer cell lines representing diverse tissue origins, such as colorectal (HT29), breast (MDA-MB-231), prostate (PC3), and pancreatic cancer (PANC-1). This observation is particularly noteworthy given that cancer cells are metabolically adaptable, utilizing amino acids not only for protein synthesis but also for nucleotide biosynthesis, energy production, and activation of cell growth-promoting signaling pathways such as mTOR [9,18]. Recent studies have further demonstrated that metabolic modulation can influence cancer stem cell metabolism and therapeutic responsiveness in pancreatic ductal adenocarcinoma, highlighting the importance of metabolic adaptation in tumor biology [19]. In addition, there is evidence that cancer cell proliferation is controlled by complex regulatory networks that extend beyond simple substrate availability [20]. The conventional hypothesis that altered amino acid availability stimulates tumor proliferation is mainly based on preclinical models, including murine models with altered amino acid ratios [21] or increased essential amino acid levels, particularly leucine [22,23,24].

Our experimental design intentionally differs from these studies. As a baseline, we utilized a standard culture medium containing 2% FCS and 20% of the amino acid concentrations typically present in standard DMEM. This composition was sufficient to support cellular growth while allowing controlled supplementation of individual amino acids. Each amino acid was then supplemented at twofold and tenfold higher concentrations than those typically used in optimized media, and left for 24 h. Under these short-term conditions, none of the 20 amino acids tested significantly stimulated proliferation in any of the four cancer cell lines examined. Furthermore, the lack of dose-dependent effects suggests that excess amino acid availability alone does not substantially modify acute tumor cell proliferation under these conditions.

Our findings should be interpreted in the context of studies reporting that certain amino acid combinations or mixtures of essential amino acids may promote tumor growth under certain experimental conditions, such as tumor auxotrophy and metabolic remodeling of cancer cells [18,25]. For example, leucine has been reported to stimulate tumor growth in breast cancer cell culture models, though clinical observations suggest that it may have both pro- and anti-tumor effects [23,24]. Many of these studies involve amino acid deprivation followed by selective supplementation, or genetically modified cancer models with altered metabolic dependencies. For instance, glutamine dependence has been demonstrated in breast cancer cell lines and in murine models. Triple-negative breast cancer cells often exhibit increased transcription of c-MYC, a transcription factor involved in glutamine metabolism. This leads to a significant increase in glutamine uptake and ultimately provides energy for tumor cell growth [26]. It has also been shown that a diet lacking serine and glycine increased survival in MYC-driven lymphoma and APC^Min/+^ induced intestinal cancer murine models [21]. These experimental contexts fundamentally differ from the clinical context of protein supplementation in cancer patients, where basic amino acid requirements are maintained, and supplementation aims to prevent catabolic muscle loss rather than disrupt tumor metabolism.

While proliferation remains largely unchanged in our short-term study, we observed subtle, non-significant variations in metabolic activity. Cysteine supplementation at high concentrations (10×) showed a slight increase in metabolic activity across all cell lines. Cysteine is not only a building block for proteins, but is also essential for glutathione synthesis, one of the most important cellular antioxidants, and thus plays a central role in oxidative stress. Since tumor cells typically contain higher levels of reactive oxygen species than healthy cells, they are more dependent on cysteine to maintain their redox balance. Several studies also show that elevated cysteine concentrations are associated with the development of various types of tumors [27]. The supplementation of very high concentrations of cysteine may therefore have influenced mitochondrial activity, potentially through effects on glutathione availability and cellular redox homeostasis. However, the observed effects in our cancer cells were not significant even at supraphysiological concentrations of cysteine.

Conversely, we observed that lysine and glutamine supplementation led to a slight numerical reduction in metabolic activity in our cancer cell lines. Although we cannot exclude effects related to the use of supraphysiological amino acid concentrations (e.g., on osmolarity or nitrogen balance), our observations regarding glutamine are in line with findings from a recent melanoma mouse model, which reported reduced cancer cell metabolism following glutamine supplementation, potentially mediated by epigenetic and metabolic reprogramming [28]. However, evidence regarding lysine supplementation and its impact on cancer cell metabolism remains limited.

The differential metabolic responses observed primarily in our PC3 prostate cancer cells following supplementation with threonine, tryptophan, and tyrosine suggest a certain degree of tissue-specificity and metabolic variability. It is known that PC3 cells exhibit distinct metabolic characteristics, including altered lipid metabolism [29] or an androgen-nonrespondent phenotype [30], which could contribute to their unique metabolic response. However, the lack of statistical significance of these metabolic variations suggests that short-term amino acid supplementation did not measurably alter metabolic activity in PC3 cells under the conditions tested. Previous studies have shown that the availability of amino acids influences the metabolism of cancer cells via nutrient sensor signaling pathways such as mTOR and AMPK, which regulate protein synthesis, cell growth, and metabolic adaptation [10,15,31]. In hepatocellular carcinoma, altered expression of amino acid transporters is associated with tumor progression, a poor prognosis, and impaired antitumor immune responses [32]. Our study focused on biologically relevant functional endpoints such as proliferation, metabolic activity, and caspase activity, rather than intracellular signaling pathways. However, future studies on nutrient-sensing pathways and the expression of amino acid transporters could provide additional mechanistic insights into cellular responses to amino acid supplementation.

In the context of apoptosis, the availability of amino acids is suggested to play an important role. Previous studies have demonstrated that an imbalance in amino acids, such as high levels of glutamate or proline, can lead to activation of apoptotic cascades and cellular dysfunctions [33]. Specific amino acid mixtures enriched in essential amino acids have been reported to promote apoptosis through autophagy activation in various cancer models [12]; on the other hand, it is also reported that amino acids such as glutamine, can support cell survival through the mTOR pathway in ovarian cancer cells [34]. These findings highlight the context-dependent effects of amino acids on apoptosis.

In our study, caspase-3/7 activity remained largely stable across the cancer cell lines following 24 h supplementation with individual amino acids. While PC3 cells showed modest variations in caspase-3/7 activity after stimulation with some amino acids (arginine, glutamine, alanine, glycine and lysine induced slight caspase activity, while cysteine, methionine, phenylalanine, tryptophan, and tyrosine showed suppressive trends), and PANC-1 cells exhibited a notable increase in caspase-3/7 activity after stimulation with high lysine concentrations, none of these changes reached statistical significance. These findings suggest that supplementation of individual amino acids, even at supraphysiological concentrations, is insufficient to activate classical apoptotic caspase pathways in the cancer cell lines studied under the experimental conditions applied.

The minimal effect of individual amino acids on caspase-3/7 activity observed in our approach contrasts with studies reporting amino acid-induced apoptosis; however, this can be explained by considering significant experimental differences. For example, studies demonstrating the pro-apoptotic effects of amino acids typically employ specific experimental conditions, such as the addition of unbalanced amino acid mixtures [12] or highly glutamine-dependent tumor cell lines [35]. Our experimental model, on the other hand, provided an adequate baseline supply of amino acids and supplemented this with individual amino acids, thereby differing substantially from models based on amino acid imbalance or deprivation. This approach was designed to mimic the increased availability of amino acids during nutritional support and may explain the limited effects of additional amino acid supplementation on caspase-3/7 activation. In cancer patients, cancer cachexia leads to profound metabolic alterations, including systemic inflammation and metabolic reprogramming that may be primarily responsible for skeletal muscle protein breakdown [36,37]. In this context, amino acids derived from dietary intake or muscle turnover may be redirected to support energy production and important physiological processes such as gluconeogenesis in the liver, immune cell function, and regulation of tumor microenvironment [38,39]. Consequently, insufficient dietary protein intake in cachectic patients may exacerbate muscle wasting and contribute to poorer clinical outcomes. Therefore, the focus of nutritional intervention for cancer patients is on ensuring adequate protein intake to counteract cancer cachexia [3]. Amino acid or protein supplementation is commonly used to support nutrition, but there are concerns that excess amino acids could be used by cancer cells to promote proliferation, metabolic activation, or apoptosis. Our findings provide evidence that the cancer cell lines studied did not exhibit measurable increases in proliferation, metabolic activity, or caspase 3/7 activity, even when exposed to very high supraphysiological concentrations of individual amino acids under short-term conditions where basic nutrient availability is maintained. However, the limited number of independent biological replicates in our study (n = 3) reduces the sensitivity of the analysis and carries the risk of overlooking subtle but biologically relevant effects. Therefore, the absence of a significant effect should be interpreted with appropriate caution. Within these limitations, the data suggest that short-term supplementation with individual amino acids to support the nutritional status of cancer patients is unlikely to directly promote the proliferation or survival of tumor cells, although longer-term effects and tumor-specific responses require further investigation.

There are various limitations of this study that should be acknowledged. First, our experiments were conducted in vitro and therefore do not fully recapitulate the complex metabolic interactions in cancer. Nutrient competition, vascular supply, hormonal regulation, and components of the cancer microenvironment, including stromal–cancer interactions, as well as infiltration of immune cells, were not considered in this study. More advanced experimental approaches, such as three-dimensional models or co-culture models incorporating cancer-associated fibroblasts and immune cells, could provide a more physiologically relevant representation of cancer biology. Second, we investigated individual amino acids only, whereas clinical nutrition interventions typically involve complex protein mixtures. Given the complexity of cancer cell metabolism, the effects of amino acid combinations cannot be excluded, since balanced mixtures of amino acids or protein hydrolysates can interact with membrane transporters and metabolic pathways, leading to cellular responses that are not observed with individual amino acids. Furthermore, it cannot be ruled out that the results may be influenced by the use of supraphysiological amino acid concentrations (e.g., glutamine at 40 mM) due to changes in osmolarity or nitrogen load. Third, the present study focused primarily on short-term proliferation, metabolic activity, and caspase-3/7 activity. High-throughput methods such as transcriptomics, proteomics or metabolomics could provide additional insights into subtle changes in gene expression, signaling pathways, and metabolic networks that may not be detectable under the experimental conditions of our study. Fourth, only four cancer cell lines of different origins were included, and the present results may not be directly applicable to other types of cancer, as the response may vary depending on tumor type and subtype with different genetic and metabolic characteristics. Finally, the observation period was limited to 24 h, and longer-term adaptations cannot be excluded.

## 5. Conclusions

In conclusion, our data suggest that supplementation with individual amino acids does not significantly affect proliferation, metabolic activity, or caspase-3/7 activity in the cancer cell lines studied under the short-term experimental conditions employed in this study. These findings contribute to the ongoing discussion regarding nutritional support in oncology and suggest that short-term exposure to individual amino acids does not measurably enhance proliferation, metabolic activity, or apoptosis-related signaling in the cancer cell lines studied. Further studies using more physiologically relevant experimental models are warranted to better understand the complex interactions between nutritional intervention and tumor metabolism. These should include long-term supplementation with amino acid mixtures or protein supplements, co-culture systems incorporating stromal and immune cells, patient-relevant organoid models, appropriate in vivo animal models of cancer cachexia, and ultimately clinical trials. These approaches would more accurately reflect the complexity of the tumor microenvironment and host-tumor metabolic interactions.

## Figures and Tables

**Table 1:** Twofold (2×) and tenfold (10×) concentrations of amino acids used in the supplementation experiments.

Amino Acid	Cat-No	2× Concentration (mM)	10× Concentration (mM)
L-Alanine	3076.1	3	15
L-Arginine monohydrochloride	1689.1	0.8	4
L-Asparagine monohydrochloride	KK 37.1	0.8	4
L-Aspartic acid	1690.1	0.30	1,5
L-Cysteine	1693.1	0.40	2
L-Glutamine	HN08.1	8	40
L-Glutamic acid	1743.1	0.3	1.5
Glycine	HN07.1	0.8	4
L-Histidine monohydrochloride	1697.1	0.2	1
L-Isoleucine	1698.1	1.6	8
L-Leucine	1699.1	1.6	8
L-Lysine monohydrate	6829.1	1.6	8
L-Methionine	1702.1	0.4	2
L-Phenylalanine	1709.1	0.8	4
L-Proline	1713.1	0.4	2
L-Serine	1714.1	0.6	3
L-Threonine	1738.1	1.6	8
Tryptophan	1739.1	0.16	0.8
Tyrosine	1741.1	0.8	4
Valine	1742.1	1.6	8

## Data Availability

The original contributions presented in this study are included in the article. Further inquiries can be directed to the corresponding author.
